# RNA-EFM: energy-based flow matching for protein-conditioned RNA sequence-structure co-design

**DOI:** 10.1093/bioadv/vbaf258

**Published:** 2025-10-22

**Authors:** Abrar Rahman Abir, Liqing Zhang

**Affiliations:** Department of Computer Science and Engineering, Bangladesh University of Engineering and Technology, Dhaka 1000, Bangladesh; Department of Computer Science, Virginia Polytechnic Institute and State University, Blacksburg, VA 24061, United States; Fralin Biomedical Research Institute at VTC: Cancer Research Center, Virginia Polytechnic Institute and State University, Washington, DC 24016, United States

## Abstract

**Motivation:**

Designing RNA molecules that can specifically bind to target proteins is fundamental to numerous biological and therapeutic applications. However, existing approaches to protein-conditioned RNA design primarily focus on structural alignment or sequence recovery, often ignoring essential biophysical factors such as molecular stability and thermodynamic feasibility.

**Results:**

To address this gap, we propose RNA-EFM, a novel deep learning framework that integrates energy-based refinement with flow matching for protein-conditioned RNA sequence and structure co-design. RNA-EFM consists of two complementary components: a flow matching objective that supervises geometric alignment between predicted and native RNA backbone structures, and an energy-based idempotent refinement that iteratively improves RNA structure predictions by minimizing both structural error and physical energy. The energy refinement is guided by biophysical priors including the Lennard-Jones potential and sequence-derived free energy, ensuring that the generated RNAs are not only geometrically plausible but also thermodynamically stable. We demonstrate the effectiveness of RNA-EFM through extensive experiments. RNA-EFM significantly outperforms state-of-the-art baselines in terms of RMSD, lDDT, sequence recovery, and binding energy improvement. These results highlight the importance of incorporating biophysical constraints into RNA design and establish RNA-EFM as a promising framework.

**Availability and implementation:**

The source code for RNA-EFM is available at: https://github.com/abrarrahmanabir/RNA-EFM.

## 1 Introduction

Ribonucleic acids (RNAs) are essential macromolecules that play multifaceted roles in various biological processes, including transcriptional and post-transcriptional gene regulation, enzymatic catalysis, and molecular recognition. Unlike proteins, RNAs possess both the ability to store genetic information and to adopt intricate secondary and tertiary structures that enable highly specific functional interactions. This structural plasticity underlies their capacity to act as dynamic regulators and sensors in cellular pathways. In recent years, this versatility has been harnessed in synthetic biology to engineer RNA-based components such as riboswitches that conditionally control gene expression and aptamers capable of binding specific protein targets with high affinity (Thavarajah *et al.* 2021, Dykstra *et al.* 2022, Nori and Jin 2024). By forming highly specific and stable interactions with proteins, RNA molecules can modulate cellular pathways, inhibit disease-associated targets, or serve as delivery agents in therapeutic settings, highlighting the growing importance of designing functional RNA-protein complexes. However, the discovery and design of functional RNAs through experimental techniques remain constrained by significant time and resource demands (Gold 2015). These limitations underscore the need for scalable computational approaches that can automate and accelerate the design of RNA sequences and structures tailored to specific functional and therapeutic outcomes (Sanchez de Groot *et al.* 2019).

Recent advances in deep learning have significantly transformed biomolecular design, especially in the context of RNA sequence and structure generation, as well as RNA attribute prediction (Abir *et al.* 2025a,b). Early computational efforts for protein-binding RNA design relied on exhaustive generation of sequences followed by structure-based filtering using thermodynamic or molecular dynamics simulations (Kim *et al.* 2010, Zhou *et al.* 2015, Buglak *et al.* 2020). Although effective, these methods were often computationally intensive and constrained by the need to pre-define structure motifs, making them less scalable for *de novo* design. Later approaches used generative models such as VAEs and LSTMs trained on SELEX data to streamline design, but these remained limited to protein-specific training and lacked generalizability to novel targets (Im *et al.* 2019, Iwano *et al.* 2022). To overcome these limitations, modern deep generative techniques have introduced geometrically-aware and structure-conditioned models. RhoFold+ (Shen *et al.* 2024) and DRfold (Li *et al.* 2023) are notable RNA three-dimensional (3D) structure prediction models, but they are not generative models. On the other hand, gRNAde proposes a graph-based autoregressive pipeline for RNA design that explicitly models 3D conformational flexibility using multi-state graph neural networks (Joshi *et al.* 2025). This method outperforms traditional tools like Rosetta in sequence recovery and speed, and demonstrates robust performance across pseudoknotted structures and dynamic ensembles. Complementarily, RNA-FrameFlow extends SE(3) equivariant flow matching to RNA backbones, addressing the challenge of RNA’s higher conformational complexity and achieving realistic 3D backbone generation through a novel frame-based representation (Anand *et al.* 2024). However, gRNAde and RNA-FrameFlow are not protein-conditioned RNA design methods. MMDiff introduces a joint SE(3)-discrete diffusion framework for the co-generation of nucleic acid and protein complexes, enabling conditional modeling of macromolecular interactions beyond isolated RNAs or proteins (Morehead *et al.* 2023). Expanding further into structure-conditioned RNA design, RNAFlow integrates inverse folding with flow matching to jointly model sequence and structure generation, leveraging fixed pre-trained structure predictors to efficiently simulate conformational ensembles (Nori and Jin 2024). This approach captures RNA dynamics and achieves superior performance in protein-conditioned design tasks. These recent developments collectively represent a paradigm shift in RNA design, from traditional, single-structure inverse folding (Zakh *et al.* 2024) toward generative frameworks that account for structural diversity, dynamics, and cross-molecular context.

While recent deep learning models have advanced the capabilities of RNA design, two critical limitations remain. First, most existing frameworks do not explicitly account for the biophysical energy landscape of RNA structures during generation. Without incorporating energy-based constraints, these models risk producing geometrically valid but energetically unstable conformations that may not fold or function as intended in biological environments. Second, the majority of current approaches rely on single-pass generation pipelines that lack mechanisms for iterative refinement, making them susceptible to accumulated prediction errors, especially for longer or more flexible RNA backbones. These limitations can significantly hinder the design of RNA molecules that must maintain stable interactions with protein targets.

In this paper, we propose RNA-EFM, a novel deep learning framework for protein-conditioned RNA sequence-structure co-design to address these limitations. Our major contributions include:

1. **Protein-conditioned RNA sequence and structure co-design:** RNA-EFM addresses the task of jointly generating RNA sequences and structures, conditioned on protein interactions, enabling the design of functional RNAs tailored to specific protein targets. Throughout all stages, both protein and RNA are consistently represented using a 3-bead coarse-grained (CG) backbone model: each RNA nucleotide is modeled by its P, C4’, and N1/N9 atoms, and each protein residue by its N, Cα, and C atoms. All model predictions and evaluation metrics are computed in this coarse-grained representation.
2. **Energy-based flow matching framework:** We propose an energy-based flow matching framework that combines flow matching with an iterative refinement process based on the idempotent constraint, ensuring the generation of accurate RNA structures that progressively align better with the target.
3. **Incorporation of biophysical signals:** To further enhance structural quality and thermodynamic stability, we integrate biophysical constraints by adding the Lennard-Jones potential and sequence-derived free energy, guiding the model toward lower-energy RNA conformations while maintaining biological relevance. To the best of our knowledge, RNA-EFM is the first method to incorporate such biophysical constraints into a flow-matching framework for RNA design.

## 2 Methods

### 2.1 Problem formulation

RNA-EFM aims to generate RNA sequences and structures conditioned on protein backbone structures, where both proteins and RNAs are represented using a 3-bead coarse-grained (CG) backbone model. Specifically, let the **target protein** backbone structure be represented as $P\in {\mathbb{R}}^{L_{p}\times 3\times 3}$, where $L_{p}$ is the number of residues, and each residue contains backbone atoms *N*, $C_{\alpha }$, and *C*. The corresponding protein sequence is $p=\{p_{i}|i=0,1,\ldots ,L_{p}-1\}$, where $p_{i}$ is a categorical token. The **RNA backbone structure** is represented as $R\in {\mathbb{R}}^{L_{r}\times 3\times 3}$, where $L_{r}$ is the number of nucleotides, and each nucleotide contains backbone atoms *P*, $C_{4}^{\prime }$, and $N_{1}/N_{9}$ (for pyrimidine and purine, respectively). The RNA sequence is $r=\{r_{i}|i=0,1,\ldots ,L_{r}-1\}$, where $r_{i}$ is a nucleotide token. We define the key distributions and entities as follows: the **prior distribution** ($p_{0}$) is a standard Gaussian over coarse-grained RNA backbone coordinates, from which a **prior RNA** backbone $R_{0}$ is sampled to serve as an initial, unstructured state. The **target distribution** ($p_{1}$) is the empirical distribution of RNA backbones observed in protein-RNA complexes, from which a **target RNA** backbone $R_{1}$ is sampled. The goal of RNA-EFM is to predict the RNA sequence r and backbone structure R, collectively referred to as the **predicted RNA**, conditioned on the given protein structure P and sequence p, so that the prediction closely matches the geometry of the target RNA. The task is thus formalized as learning a mapping from (P,p) and a sampled prior RNA backbone $R_{0}$ to a predicted RNA sequence and structure (r,R) that are jointly compatible with the target protein.

### 2.2 Overview of the RNA-EFM

**Figure 1.**
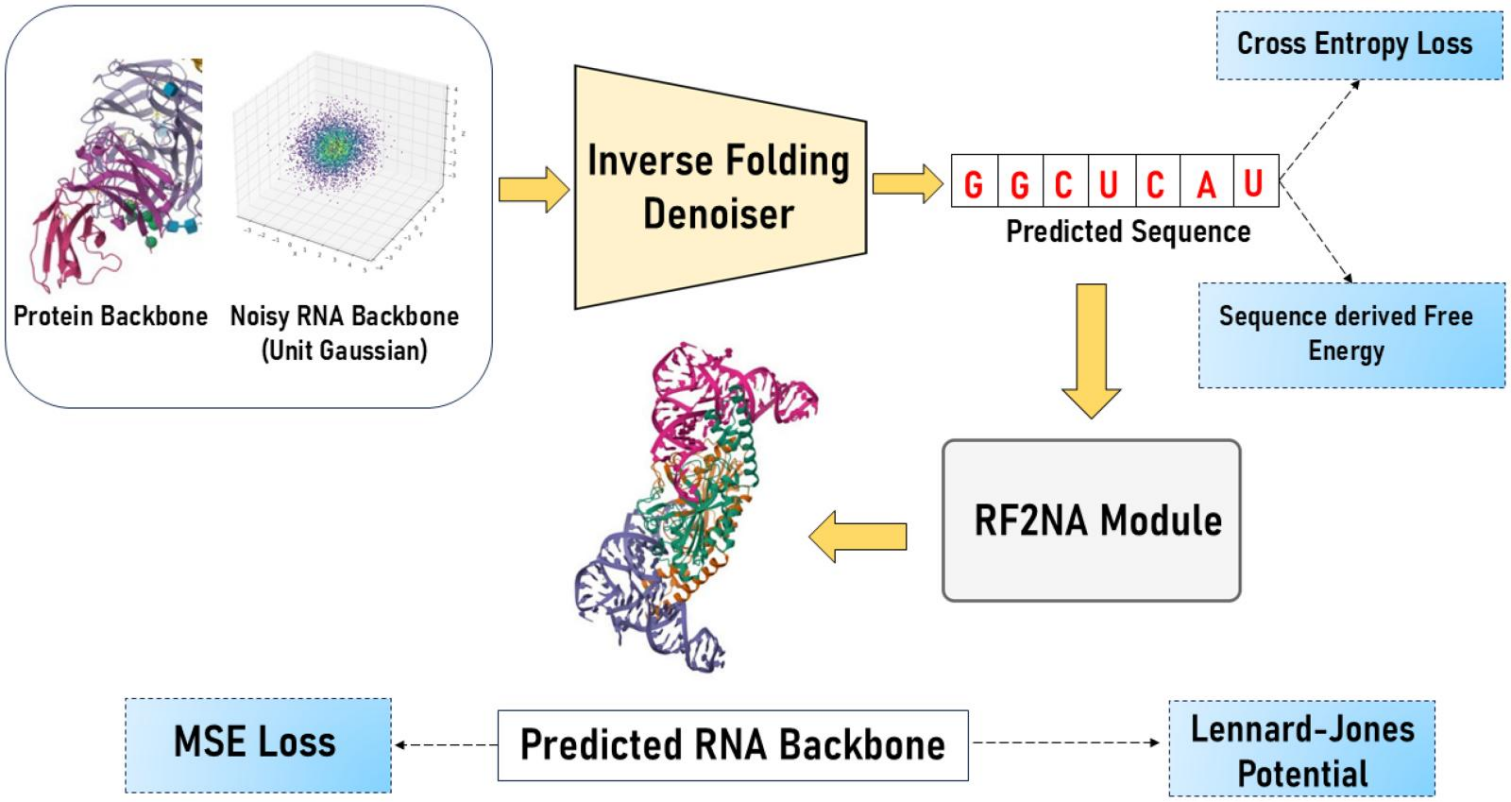
Overview of the RNA-EFM architecture. The model takes a protein backbone and a noisy RNA backbone as inputs and then the inverse folding denoiser predicts the RNA sequence and this predicted sequence is passed to the pretrained RF2NA module to predict the RNA backbone structure and the predicted RNA backbone is refined iteratively by minimizing the mean squared error (MSE) loss and incorporating the Lennard-Jones potential for structural stability and the predicted sequence is optimized using a combination of sequence derived free energy and cross-entropy loss, ensuring biophysically plausible and structurally accurate RNA generation.

RNA-EFM is a protein-conditioned deep learning framework for the joint design of RNA sequences and structures that can bind target proteins. In this work, both protein and RNA are consistently represented using a 3-bead coarse-grained (CG) backbone model, where each RNA nucleotide is modeled by its P, C4’, and N1/N9 atoms, and each protein residue by its N, Cα, and C atoms. All model predictions and evaluations are conducted in this coarse-grained space. Our approach combines the principles of flow matching and energy-based refinement to generate biologically plausible RNA structures. The flow matching objective aligns the predicted coarse-grained structures with the target RNA backbone distribution, ensuring accurate geometric correspondence. Additionally, an iterative refinement process integrates biophysical energy constraints, leveraging a combination of sequence-derived free energy and physically motivated interatomic potentials. This approach enables the model to design RNA structures that are not only geometrically aligned with experimental targets but also energetically stable and biologically meaningful. The architecture of RNA-EFM is shown in Fig. 1. Next, we briefly describe the flow matching objective, the energy-based idempotent refinement process, and the training and inference procedures of RNA-EFM.

### 2.3 Flow matching objective

The RNA-EFM framework adopts flow matching to transform a prior distribution $p_{0}(R)$ into a target distribution $p_{1}(R)$, where R represents RNA backbone structures following (Nori and Jin 2024). This transformation is achieved by parameterizing the flow as a sequence of time-dependent conditional probability distributions $p_{t}(R_{t}|R_{1})$, where t∈[0,1] denotes the interpolation step.

The intermediate RNA backbone structure $R_{t}$ at any time step *t* is obtained through linear interpolation between a sample from the prior distribution $R_{0}∼p_{0}(R)$ and a sample from the target distribution $R_{1}∼p_{1}(R)$, given by $R_{t}|R_{1}=(1-t)R_{0}+tR_{1}$, where t∼U(0,1) is uniformly sampled. This interpolation constructs a continuous path from $p_{0}$ to $p_{1}$ that the model learns to approximate. To account for the geometric properties of RNA backbone structures, we utilize the Kabsch algorithm to align the prior sample $R_{0}$ with the target structure $R_{1}$. The Kabsch algorithm minimizes the root-mean-square deviation (RMSD) between two sets of points, ensuring invariance to rigid transformations such as rotation and translation. The aligned structure is given by $R_{0}{}^{*}=K(R_{0},R_{1})$, where K(·,·) denotes the Kabsch alignment operation. The flow matching objective aims to minimize the discrepancy between the true and predicted vector fields that govern the transformation from $p_{0}$ to $p_{1}$. The true vector field $v_{t}(R_{t}|R_{1})$ is defined as:


(1) $$v_{t}(R_{t}|R_{1})=\frac{R_{1}-R_{t}}{1-t},$$

while the predicted vector field, parameterized by the neural network $f(R_{t};\theta )$ which predicts RNA backbone from a noisy intermediate backbone, is expressed as:


(2) $${\hat{v}}_{t}(R_{t};\theta )=\frac{f(R_{t};\theta )-R_{t}}{1-t}.$$

The flow matching loss $L_{\mathrm{FM}}$ is formulated as the expected squared difference between these vector fields:


(3) $$L_{\mathrm{FM}}=E_{R_{1}∼p_{1},R_{t}∼p_{t}}>[∥{\hat{v}}_{t}(R_{t};\theta )-v_{t}(R_{t}|R_{1}){∥}^{2}>].$$

Substituting (1) and (2) into (3), we derive:


(4) $$L_{\mathrm{FM}}=E_{R_{1},R_{t}}>[\frac{1}{{\left(1-t\right)}^{2}}∥f(R_{t};\theta )-R_{1}{∥}^{2}>].$$

Finally, incorporating the Kabsch alignment to ensure invariance to rotational and translational transformations, the objective becomes:


(5) $$L_{\mathrm{FM}}=E_{R_{1},R_{t}}>[\frac{1}{{\left(1-t\right)}^{2}}∥f(R_{t};\theta )-K(R_{1},R_{t}){∥}^{2}>].$$

After marginalizing over *t*, the final loss reduces to the mean squared error (MSE) between the aligned predicted and ground truth backbone structures:


(6) $$L_{\mathrm{FM}}=\mathrm{MSE}({\hat{R}}_{1},K(R_{1},{\hat{R}}_{1})).$$

This formulation ensures that the RNA backbone predictions align accurately with the target structures while preserving geometric invariance.

### 2.4 Energy-based idempotent flow matching in RNA-EFM

To improve the biological plausibility of predicted RNA structures, RNA-EFM incorporates an energy-based refinement framework following (Zhou et al. 2015), combining flow matching with biophysical constraints derived from RNA structure and sequence. This ensures that predicted structures align both geometrically and energetically with the target. The core idea is to iteratively refine the predicted RNA backbone structures by minimizing an energy function that penalizes deviations from the target structure while incorporating physical energy constraints for stability. The refinement is governed by the conditional probability distribution:


(7) $$p({\hat{R}}_{1}|R_{1})\propto \;\text{exp}\;>(-\frac{1}{2\sigma^{2}}∥{\hat{R}}_{1}-R_{1}{∥}^{2}-\alpha U({\hat{R}}_{1},\hat{r})>)$$

where ${\hat{R}}_{1}$ is the predicted RNA backbone structure, $\hat{r}$ is the predicted RNA sequence, $R_{1}$ is the target structure, and $U({\hat{R}}_{1},\hat{r})$ represents the physical energy term combining the Lennard-Jones potential and the free energy approximated by a differentiable neural network trained on Vienna RNAfold outputs (Lorenz *et al.* 2011). The Lennard-Jones potential is defined as:


(8) $$U_{\text{LJ}}(d)=4\epsilon >[{>(\frac{\sigma }{d}>)}^{12}-{>(\frac{\sigma }{d}>)}^{6}>]$$

where *d* is the distance between two atoms, σ is the distance at which the potential energy is zero, and ϵ is the depth of the potential well, which determines the strength of the interaction. The equilibrium distance, where attractive and repulsive forces are balanced and the force is zero, occurs at $d=\sigma \times 2^{1/6}$. In all experiments, the Lennard-Jones potential was computed using ϵ=0.2 and σ=3.5 Å. We acknowledge that this simple coarse-grained potential cannot capture all details of atomic interactions. However, our rigorous experiments and ablation study show that this simplified energy model can greatly enhance the performance. The Lennard-Jones potential effectively eliminates steric clashes in generated structures, as validated by inter-bead distance analysis (Section 4, available as supplementary data at *Bioinformatics Advances* online).Taking the negative logarithm of the probability distribution in (7) results in the energy function, which combines the geometric loss and biophysical energy term:


(9) $$E({\hat{R}}_{1})=\frac{1}{2\sigma^{2}}∥{\hat{R}}_{1}-R_{1}{∥}^{2}+\alpha U({\hat{R}}_{1},\hat{r})$$

which is minimized during refinement. The gradient, used for iterative refinement, is given by:


(10) $$\nabla_{{\hat{R}}_{1}}E({\hat{R}}_{1})=\frac{1}{\sigma^{2}}({\hat{R}}_{1}-R_{1})+\alpha \nabla_{{\hat{R}}_{1}}U({\hat{R}}_{1},\hat{r})$$

The refinement process is guided by the idempotent flow matching property, ensuring stabilization of predicted structures through repeated refinement until convergence. Mathematically, it is expressed as:


(11) $${\hat{R}}_{1}{}^{*}=f({\hat{R}}_{1}{}^{*};\theta ),$$

where f(·;θ) denotes the neural network predictor. This fixed-point formulation means that the refinement operator converges to a stable solution ${\hat{R}}_{1}{}^{*}$ when applied recursively:


$${\hat{R}}_{1}^{\left(k+1\right)}=f({\hat{R}}_{1}^{\left(k\right)};\theta ),$$

and in the limit k→∞, we have ${\hat{R}}_{1}^{*}=f({\hat{R}}_{1}^{*};\theta )$. Under mild conditions (e.g. if *f* is a contraction mapping near the optimum), convergence to a fixed point is guaranteed by the **Banach fixed-point theorem**. This theoretically justifies the stabilization observed in practice. Furthermore, the energy function in (9) is minimized by f(·;θ) during training, which encourages the predictor to map its own output to itself if it already lies near a local energy minimum, i.e.


$${\hat{R}}_{1}^{*}=\arg\underset{{\hat{R}}_{1}}{\min}E({\hat{R}}_{1}).$$

Thus, idempotency and energy minimization are tightly coupled: idempotency ensures that the prediction is stable and fixed under the refinement mapping, and the energy term ensures that this fixed point corresponds to a biophysically plausible structure. The training objective incorporates both flow matching and biophysical energy constraints, defined as:


(12) $$L_{\text{ID}}=E_{t∼U(0,1)}E_{R_{t}∼p_{t}}>[∥f({\hat{R}}_{1};\theta )-R_{1}{∥}^{2}+\beta U({\hat{R}}_{1},\hat{r})>]$$

where the first term minimizes deviation from the target structure while the second term penalizes high-energy configurations. Minimizing this objective ensures RNA-EFM generates both geometrically accurate and energetically favorable RNA structures.

Algorithm 1 RNA-EFM Training Algorithm

**Require:** Prior distribution $p_{0}$, Target distribution $p_{1}$, Maximum refinement steps $K_{\text{max}}$, Weighting parameter β

 1: **while** Training **do** 

 2:    Sample prior RNA backbone $R_{0}∼N{\left(0,I_{3}\right)}^{L_{r}}$

 3:    Sample target RNA sequence and backbone (r, $R_{1})∼p_{1}$

 4:    Align $R_{0}$ with $R_{1}$ using Kabsch to obtain $R_{0}^{*}$

 5:    Sample timestep t∼Uniform[0,1]

 6:    Compute interpolation: $R_{t}\leftarrow t\cdot R_{1}+(1-t)\cdot R_{0}^{*}$

 7:    Predict RNA sequence and structure:

 8:    $\left(\hat{r},{\hat{R}}_{1})\leftarrow f(R_{t};\theta \right)$

 9:    Sample decision variable m∼Uniform[0,1]

 10:    **if**  m≤0.5  **then** 

 11:     Sample refinement steps $k∼\text{randint}(1,K_{\text{max}})$

 12:     Initialize refinement list: ${\hat{R}}_{1}^{\text{list}}\leftarrow \emptyset$

 13:     **for**  i=0 to *k* **do** 

 14:      ${\hat{R}}_{1}\leftarrow f({\hat{R}}_{1};\theta )$

 15:      ${\hat{R}}_{1}^{\text{list}}\leftarrow {\hat{R}}_{1}^{\text{list}}\cup {\hat{R}}_{1}$

 16:     **end for** 

 17:     Compute refinement loss:

 18:     $L_{\text{ID}}\leftarrow \frac{1}{\left|{\hat{R}}_{1}^{\text{list}}\right|}\sum_{{\hat{R}}_{1}\in {\hat{R}}_{1}^{\text{list}}}∥{\hat{R}}_{1}-R_{1}{∥}^{2}$

      $+\beta U({\hat{R}}_{1},\hat{r})+\text{CrossEntropy}(r,\hat{r})$

 19:    **else** 

 20:     Compute flow matching loss:

 21:     $L_{\text{FM}}\leftarrow ∥{\hat{R}}_{1}-R_{1}{∥}^{2}$

      $+\text{CrossEntropy}(r,\hat{r})$

 22:    **end if** 

 23:    Update model parameters θ

 24: **end while** 

 25: **return** Structure predictor $f(R_{t};\theta )$

### 2.5 Training and inference of RNA-EFM

In RNA-EFM, the training process is designed to jointly optimize RNA sequence and structure prediction under protein conditioning. Each training iteration begins by sampling a random RNA backbone from a Gaussian prior and selecting a target RNA, consisting of both sequence and structure, from the experimentally determined training dataset. The initial backbone is aligned to the target structure using the Kabsch algorithm, ensuring that the subsequent geometric interpolation is performed in a common coordinate system. A random timestep *t* is then drawn, and a linear interpolation between the aligned prior and the target backbone produces an intermediate RNA backbone $R_{t}$. At this stage, similar to RNA-Flow (Nori and Jin 2024), the model first predicts an RNA sequence using an inverse folding denoiser module; this is followed by generating the corresponding RNA structure via a structure predictor RF2NA [RoseTTAFold2NA (Baek *et al.* 2024)]. As the inverse folding denoiser, we adopt the Noise-to-Seq module from RNA-Flow (Nori and Jin 2024), a graph-based RNA inverse folding model that autoregressively predicts RNA sequences from noised backbone structures. It operates on a protein-RNA complex represented as a graph G=(V,E), where nodes correspond to protein $C_{\alpha }$ and RNA $C_{4}^{\prime }$ atoms, and edges connect spatial neighbors. Each node is enriched with geometric and biochemical features, while edge features capture distances and relative orientations. The model uses a GVP-based encoder (Jing *et al.* 2020) to process the protein-RNA graph and a decoder to predict nucleotide probabilities. A cross entropy loss is applied between the predicted and true sequences, ensuring that sequence accuracy is directly supervised. The training objective is split into two alternating phases. In 50% of iterations, the model minimizes a flow matching loss, which penalizes the mean squared error between the predicted RNA backbone and the target structure. This phase focuses on establishing geometric alignment. In the remaining 50% of iterations, the predicted structure is refined through an iterative process. Specifically, the model repeatedly feeds its own output back into the structure predictor. This iterative refinement enforces an idempotent property, meaning that once the predictions converge, further applications of the predictor produce minimal change. The loss during this refinement phase not only includes the mean squared error with respect to the target but also integrates an energy term—derived from biophysical constraints such as the Lennard-Jones potential and sequence-derived free energy—which discourages energetically unfavorable conformations. By alternating between these two training phases, RNA-EFM effectively learns to generate RNA designs that are both geometrically accurate and energetically stable in the context of protein binding. Algorithm 1 describes the training process. Hyperparameters for RNA-EFM can be found in the Supplementary Material, available as supplementary data at *Bioinformatics Advances* online. However, users of RNA-EFM may also adopt Lennard-Jones potential parameters from (Yangaliev and Ozkan 2025), which provides well-validated bead definitions and interaction parameters for RNA structures. All experiments were performed on NVIDIA RTX A4500 GPU. The total training time for RNA-EFM is approximately 14 hours, while RNAFlow requires about 12 hours on the same hardware. The additional training time for RNA-EFM is due to the idempotent refinement loss. Importantly, the inference time per test sample remains virtually unchanged, with both RNA-EFM and RNAFlow requiring approximately 11 seconds per sample. Notably, RNA-EFM achieves state-of-the-art performance with no practical increase in inference time.

During inference, RNA-EFM only requires as input the protein sequence and its backbone coordinates. The model first uses the Noise to Seq module to predict an RNA sequence compatible with the protein target. This predicted sequence, together with the protein structure, is then passed to the RF2NA module, which generates the corresponding RNA backbone coordinates in a coarse grained format. Both modules operate in a single forward pass without iterative refinement. The outputs of inference are the designed RNA sequence and its predicted backbone structure, each geometrically aligned to the protein and optimized for biophysical plausibility.

## 3 Experiments

### 3.1 Dataset

We use the same dataset and preprocessing steps as RNAFlow (Nori and Jin 2024), derived from the 2020 release of the PDBBind database (Liu *et al.* 2017), consisting of protein–RNA complexes. Following RNAFlow, we evaluate our model on two types of dataset splits:

1. **RF2NA split:** To avoid test-time data leakage from the RF2NA structure predictor, we assign all complexes from the RF2NA validation and test sets to our test split. The remaining data is split into training and validation sets using a 9:1 ratio.
2. **RNA sequence similarity split:** To evaluate generalization to unseen RNA sequences, we cluster all RNA chains using CD-HIT (Fu *et al.* 2012) with an 80% sequence identity threshold, as in (Joshi *et al.* 2025). The clusters are then split into train, validation, and test using an 8:1:1 ratio.

Table 1 presents the statistics of the dataset.

**Table 1.** Number of protein–RNA complexes in each split.

Split type	Train	Validation	Test
RF2NA-aware split	1059	117	16
Sequence similarity split	1015	105	72

### 3.2 Baselines

To evaluate RNA-EFM, we compare it against multiple baselines for RNA structure and sequence generation. For fair comparison, we only choose the models developed for protein conditioned RNA sequence-structure co-design, which is the central objective of this study. For structure generation, we consider **Conditional MMDiff** (Morehead *et al.* 2023), an SE(3)-equivariant diffusion model, and **RNAFlow** (Nori and Jin 2024), a flow matching-based framework, along with its variants. **RNAFlow-Base**, initializes structure generation using RF2NA by folding a mock RNA sequence composed entirely of adenines and iteratively refining the predicted conformation, while **RNAFlow-Traj** conditions on multiple RNA backbone conformations. **RNAFlow-Base + Rescore** and **RNAFlow-Traj + Rescore** further enhance selection through a rescoring model. For sequence generation, we include a **Random baseline**, which selects nucleotides uniformly, an **LSTM-based model** that autoregressively predicts RNA sequences (Im *et al.* 2019), **Conditional MMDiff** and **RNAFlow** along with its variants.

### 3.3 Evaluation metrics

We evaluate structure generation using **Root Mean Square Deviation (RMSD)** and **local Distance Difference Test (lDDT)**, both computed after alignment using the Kabsch algorithm. RMSD measures overall structural deviation, while lDDT captures local atomic accuracy. For sequence generation, we report both the **recovery rate** (in main results), defined as the percentage of nucleotides correctly predicted with respect to the ground-truth RNA sequence, and the **Levenshtein distance** (in Table 2, available as supplementary data at *Bioinformatics Advances* online), which measures the minimum number of single-nucleotide edits (insertions, deletions, or substitutions) required to transform the predicted sequence into the ground-truth sequence. Additionally, we compute the **Improved Binding Percentage (IMP)**, which measures the percentage of designed RNA–protein complexes that exhibit lower (i.e. better) binding free energy ΔG compared to the original. Binding energies are estimated using the Rosetta scoring function, implemented by PyRosetta (Chaudhury *et al.* 2010).

### 3.4 Performance evaluation

We comprehensively evaluate RNA-EFM on both structure and sequence generation tasks.

#### 3.4.1 Structure generation

RNA-EFM achieves the best overall performance in structure prediction across both evaluation splits, as shown in Table 2. On the RF2NA-aware split, RNA-EFM attains an RMSD of 10.00 and an lDDT of 0.60, significantly outperforming strong baselines such as RNAFlow-Base (RMSD 12.85, lDDT 0.51) and RNAFlow-Traj (RMSD 13.12, lDDT 0.52). The most competitive baseline, RNAFlow-Base + Rescore, yields an RMSD of 10.61 and lDDT of 0.53, yet RNA-EFM surpasses it with a 5.75% reduction in RMSD and a 13.21% improvement in lDDT. On the sequence similarity split, RNA-EFM maintains its advantage with an RMSD of 13.00 and lDDT of 0.60, improving upon RNAFlow-Base + Rescore (RMSD 14.60, lDDT 0.56) by 10.96% in RMSD and 5.26% in lDDT. These results highlight RNA-EFM’s robustness in generating structurally accurate RNA backbones even under distributional shifts in sequence space.

**Table 2.** RNA structure generation results where we report mean ± standard error of the mean (SEM) for RMSD and lDDT metrics and best values are shown in bold.

Method	RF2NA split		Sequence similarity split	
	RMSD	lDDT	RMSD	lDDT
Conditional MMDiff	14.82 ± 1.01	0.34 ± 0.02	17.42 ± 0.86	0.38 ± 0.01
RNAFlow-Base	12.85 ± 0.63	0.51 ± 0.01	14.77 ± 0.34	0.57 ± 0.01
RNAFlow-Traj	13.12 ± 0.64	0.52 ± 0.01	15.11 ± 0.33	0.57 ± 0.00
RNAFlow-Base + rescore	10.61 ± 1.73	0.53 ± 0.03	14.60 ± 1.05	0.56 ± 0.02
RNAFlow-Traj + rescore	15.30 ± 1.89	0.52 ± 0.03	15.31 ± 0.93	0.56 ± 0.02
**RNA-EFM**	**10.00** ± **0.50**	**0.60** ± **0.01**	**13.00** ± **0.40**	**0.60** ± **0.01**

#### 3.4.2 Sequence generation

For sequence recovery, RNA-EFM again outperforms all baselines, as shown in Table 3. On the RF2NA split, RNA-EFM achieves a recovery rate of 0.40, while the best competing method, RNAFlow-Traj + Rescore, reaches 0.37. Similarly, on the sequence similarity split, RNA-EFM yields a recovery of 0.35, compared to 0.32 from RNAFlow-Base + Rescore. Other generative baselines such as RNAFlow-Traj (0.31) and Conditional MMDiff (0.24) show notably lower performance. These improvements—8.11% and 9.38% over the strongest baselines in each split—underscore RNA-EFM’s effectiveness in accurately generating biologically meaningful RNA sequences conditioned on protein structure.

**Table 3.** RNA sequence generation results where we report mean ± standard error of the mean (SEM) for native sequence recovery and best values are shown in bold.

Method	RF2NA split	Sequence Similarity split
Random	0.25 ± 0.00	0.25 ± 0.00
LSTM	0.27 ± 0.01	0.24 ± 0.01
Conditional MMDiff	0.24 ± 0.02	0.22 ± 0.02
RNAFlow-Base	0.30 ± 0.02	0.30 ± 0.01
RNAFlow-Traj	0.31 ± 0.01	0.28 ± 0.01
RNAFlow-Base + Rescore	0.33 ± 0.02	0.32 ± 0.03
RNAFlow-Traj + Rescore	0.37 ± 0.05	0.29 ± 0.02
**RNA-EFM**	**0.40** ± **0.03**	**0.35** ± **0.02**

#### 3.4.3 Energy evaluation

**Figure 2.**
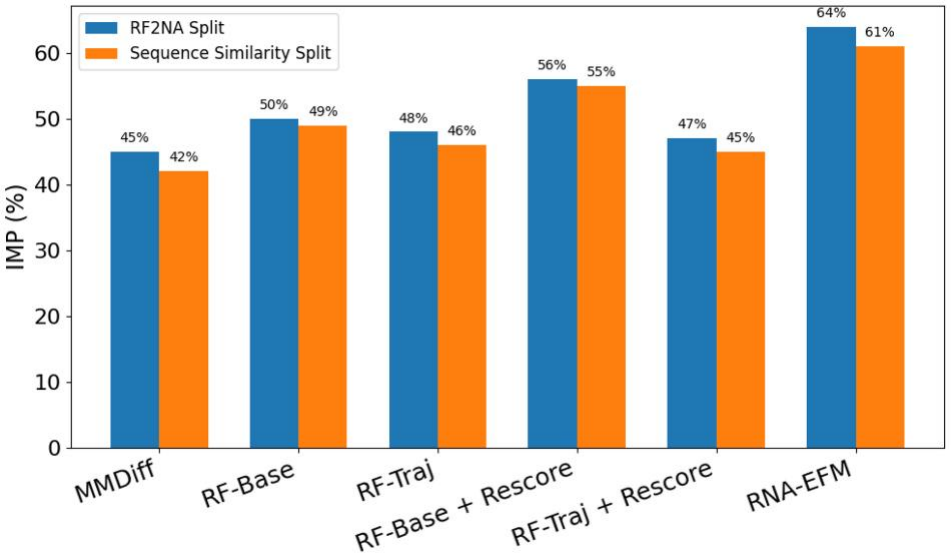
Comparison of improved binding percentage (IMP) across different models. Comparison of IMP across different models where RNA-EFM achieves the highest IMP across both the RF2NA pre-training and sequence similarity splits, indicating superior design of energetically favorable RNA–protein complexes and RF = RNAFlow.

To assess the thermodynamic favorability of designed RNA–protein complexes, we evaluate the **IMP**. For this experiment, we generate 50 samples per complex and compute the percentage of those that outperform the native complex in binding energy. Figure 2 presents the IMP scores across both the RF2NA split and the sequence similarity split. RNA-EFM achieves the highest IMP in both settings, with 64% on the RF2NA split and 61% on the sequence similarity split. These results indicate that a substantial majority of RNA-EFM’s designs lead to enhanced binding affinity compared to native structures. The next best performer, RNAFlow-Base + Rescore, attains 56% and 55% respectively, while other methods such as Conditional MMDiff, RNAFlow-Base, and RNAFlow-Traj yield considerably lower IMP values, often below 50%. These findings demonstrate that RNA-EFM not only excels at generating accurate RNA sequences and structures, but also significantly improves the likelihood of producing biologically functional, high-affinity RNA–protein complexes.

Together, these results highlight the efficacy of RNA-EFM’s dual objectives: flow matching ensures geometric alignment, while energy-based refinement yields biologically plausible and thermodynamically favorable designs. RNA-EFM consistently outperforms prior methods across all evaluation axes—structure, sequence, and binding energy—demonstrating its strength as a unified framework for protein-conditioned RNA design. We have also reported performance comparison on an independent test set with 73 complexes in the Table 1, available as supplementary data at *Bioinformatics Advances* online.

#### 3.4.4 Sequence and structure novelty

To assess the generalizability of generated RNAs, we evaluate both sequence and structure novelty. For each generated RNA sequence $\hat{r}$, we compute sequence recovery with respect to the most similar training sequence as


$$\text{recovery}(\hat{r})=\underset{r_{\text{train}}}{\max}\frac{1}{L_{r}}\sum_{i=1}^{L_{r}}I>[{\hat{r}}_{i}={\left(r_{\text{train}}\right)}_{i}>],$$

and define sequence novelty as $1-\text{recovery}(\hat{r})$. For structure novelty, we compute the maximum TM-score between each generated RNA structure $\hat{R}$ and all training structures, and define structure novelty as $1-\text{TM-score}(\hat{R})$. Higher novelty values indicate greater dissimilarity to the training set.

**Figure 3.**
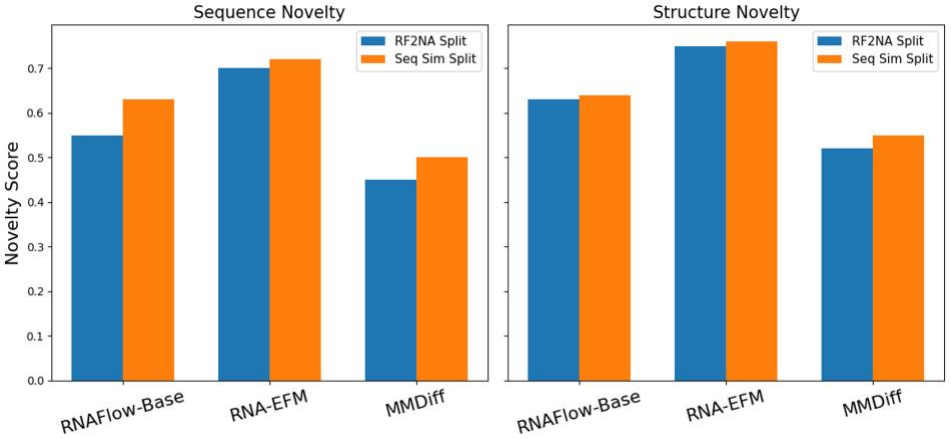
Comparison of sequence and structure novelty across different models. Comparison of sequence and structure novelty across different models where RNA-EFM consistently outperforms RNAFlow-Base and MMDiff on both the RF2NA and sequence similarity splits, demonstrating its superior ability to generate novel RNA sequences and structures.

As shown in Fig. 3, RNA-EFM achieves the highest novelty scores in both sequence and structure categories across the RF2NA and sequence similarity splits, with sequence novelty reaching up to 0.72 and structure novelty up to 0.76. This indicates that RNA-EFM not only learns to generate accurate and functional RNAs but also avoids overfitting to training examples. In contrast, MMDiff produces the least novel RNAs in both structure and sequence space. RNAFlow-Base lies in between, demonstrating moderate novelty. These results emphasize the superior capacity of RNA-EFM to generalize and produce structurally and sequentially diverse RNA designs.

## 4 Ablation study

**Figure 4.**
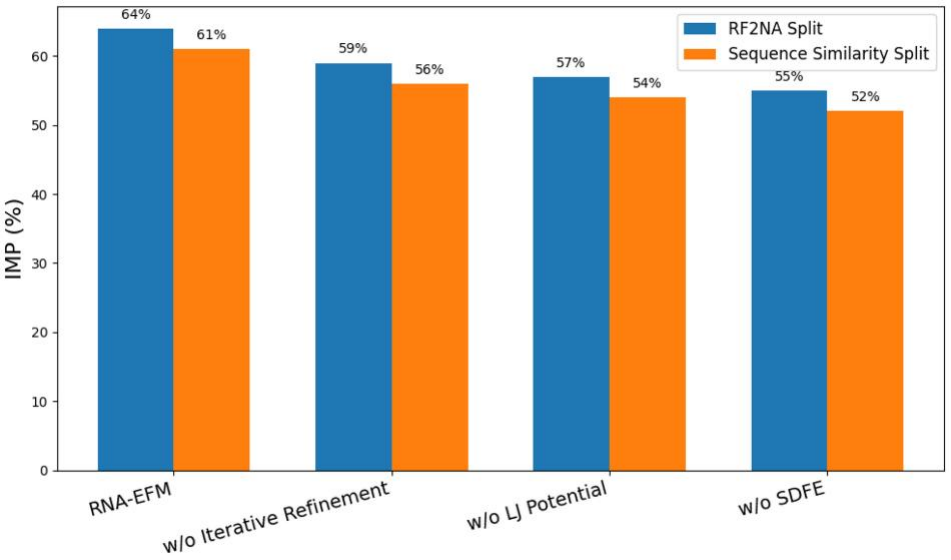
Ablation analysis of RNA-EFM on the RF2NA and sequence similarity splits using IMP (% of designed RNA–protein complexes with lower binding energy than native). Ablation analysis of RNA-EFM on the RF2NA and sequence similarity splits using IMP (% of designed RNA–protein complexes with lower binding energy than native) where we examine the impact of removing three key components: iterative refinement, the Lennard-Jones (LJ) potential, and sequence-derived free energy (SDFE) and RNA-EFM outperforms all ablated variants, highlighting the importance of each component in guiding the model toward energetically favorable RNA structures.

**Figure 5.**
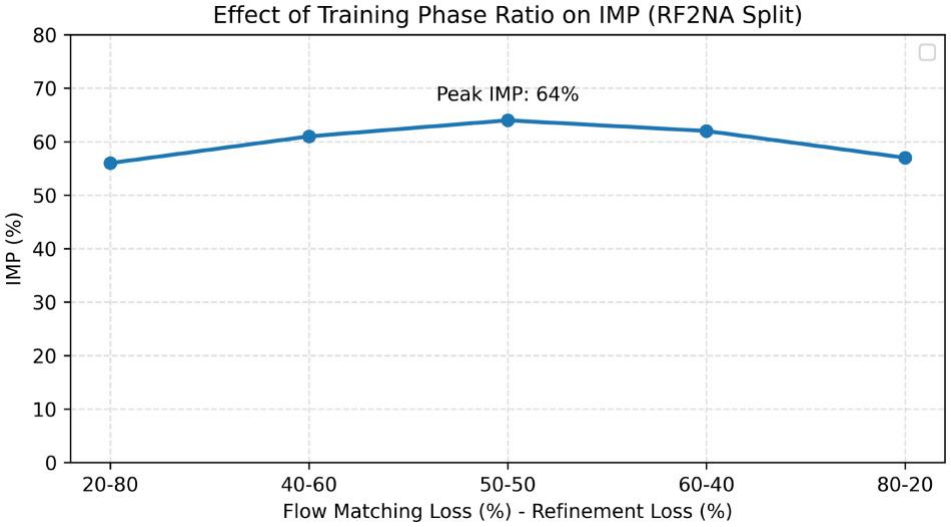
Effect of training phase ratio on model performance. Effect of training phase ratio on model performance where IMP (%) values on the RF2NA split are shown for different allocations between flow matching loss and refinement loss during training and Peak performance is achieved at a balanced 50–50 ratio, demonstrating the necessity of both objectives for optimal design.

To evaluate the contribution of each core component in RNA-EFM, we conduct an ablation study on the RF2NA and sequence similarity splits using the IMP metric, which quantifies the percentage of generated RNA–protein complexes with improved (i.e. lower) binding free energy compared to their native counterparts. We analyze three variants: (i) RNA-EFM without iterative refinement, (ii) without the Lennard-Jones (LJ) potential, and (iii) without sequence-derived free energy (SDFE). As shown in Fig. 4, removing any of these components consistently reduces performance. Notably, discarding iterative refinement leads to a 5% absolute drop in IMP on both splits, underscoring its role in driving structural convergence. Omitting LJ potential or SDFE also causes a notable decline, demonstrating that both atomic-level and sequence-level biophysical constraints contribute to improved binding stability. In addition to the single-component ablations, we also evaluated a variant of RNA-EFM with all three novel contributions—iterative refinement, Lennard-Jones potential, and sequence-derived free energy removed simultaneously. This variant reduces to the same architecture and objective as RNAFlow. As expected, its performance closely matches that of RNAFlow, confirming that the observed improvements are specifically attributable to the novel components introduced in RNA-EFM rather than other implementation details. This further supports the necessity and effectiveness of integrating all three biophysical and refinement mechanisms in our method. Finally, to further investigate the impact of training phase balance, we conduct an ablation study varying the ratio between flow matching loss and refinement loss during training. As shown in Fig. 5, we experiment on ratios ranging from 20–80 to 80–20. The results reveal that model performance, as measured by the IMP metric on the RF2NA split, peaks at a 50–50 split between flow matching and refinement, indicating that effective design requires a balanced contribution from both geometric alignment and biophysical refinement objectives. This finding empirically justifies our default choice of a 50–50 ratio for optimal model performance.

## 5 Case study

**Figure 6.**
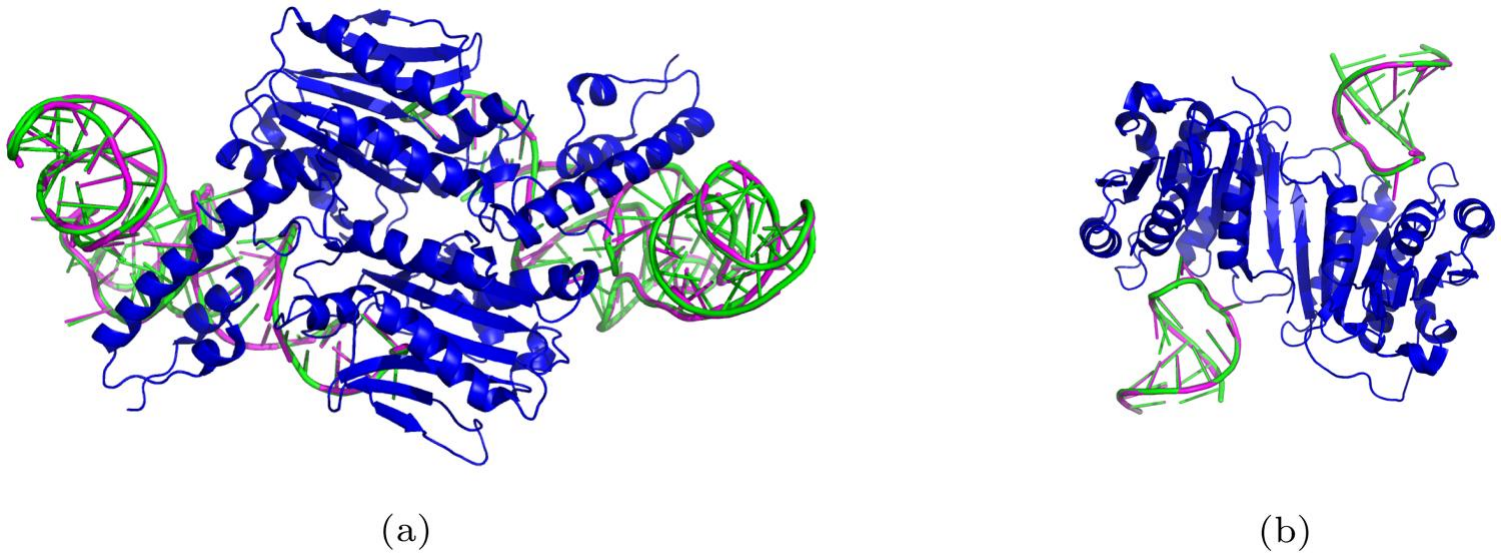
Superimposition of ground-truth and predicted RNA backbones within the protein–RNA complexes. Superimposition of ground-truth and predicted RNA backbones within the protein–RNA complexes for (a) PDB 2ZNI and (b) PDB 8H1B and the protein is shown in blue, ground-truth RNA backbones in green, and predicted RNA backbones in magenta and this visualization highlights the structural consistency between the ground-truth and generated conformations.

To further demonstrate the effectiveness of RNA-EFM in generating accurate RNA structures conditioned on protein targets, we conducted a detailed case study on two representative RNA–protein complexes: PDB 2ZNI and PDB 8H1B. We used the predicted RNA sequences and backbones generated by RNA-EFM and compared them with the experimentally resolved true structures from the corresponding PDB entries. Structural alignment and visualization were performed using PyMOL (Schrodinger LLC 2015). In particular, we superimposed the predicted RNA structures onto the ground-truth complexes while keeping the protein chains fixed. For visual clarity, proteins were colored blue, ground-truth RNA backbones were rendered in green, and predicted RNA backbones in magenta. Superimposed images are shown in Fig. 6. This allowed us to qualitatively assess how closely the generated RNA structures align with native conformations. Additionally, we computed RMSD between ground-truth and predicted RNA backbone atoms using the Kabsch algorithm, along with sequence recovery and binding energy improvement (IMP) using PyRosetta. These quantitative evaluations are reported in Table 4. RNA-EFM achieves the lowest RMSD and highest sequence recovery and IMP across both examples, supporting its ability to generate biologically and structurally meaningful RNA designs conditioned on proteins.

**Table 4.** Evaluation metrics on PDB 2ZNI and PDB 8H1B where we report RMSD (lower is better), sequence recovery (%), and IMP (%) for each method and best values are shown in bold.

Method	2ZNI			8H1B		
	RMSD ↓	Seq. Rec. ↑	IMP ↑	RMSD ↓	Seq. Rec. ↑	IMP ↑
RNA-EFM	**2.93**	**54%**	**72%**	**4.78**	**46%**	**64%**
RNAFlow	3.40	49%	67%	5.10	42%	58%
MMDiff	5.10	48%	54%	6.20	39%	50%

## 6 Conclusion

In this work, we present RNA-EFM, a novel deep learning framework for RNA sequence and structure generation conditioned on protein interactions. By integrating biophysical energy-based refinement with idempotent flow matching, RNA-EFM generates RNA structures that are not only geometrically accurate but also energetically stable and biologically relevant. Our experiments demonstrate that RNA-EFM consistently outperforms existing baselines across multiple evaluation settings, including structure prediction, sequence recovery, and thermodynamic plausibility. As a future direction, expanding the framework to support multi-protein binding or RNA aptamer design for small molecules opens promising avenues for broader applications in therapeutic RNA engineering.

## Supplementary Material

vbaf258_Supplementary_Data

## Data Availability

The source code and dataset for RNA-EFM can be found at: https://github.com/abrarrahmanabir/RNA-EFM.
