# Supplementary material for: RNA-EFM: energy-based flow matching for protein-conditioned RNA sequence-structure co-design
Source: Bioinform Adv. 2025 Oct 22;5(1):vbaf258. doi: 10.1093/bioadv/vbaf258 (PMC12701795; doi:10.1093/bioadv/vbaf258)
Supplement: vbaf258_Supplementary_Data [file vbaf258_supplementary_data.pdf]

Supplementary Materials for

RNA-EFM : Energy Based Flow Matching for Protein-conditioned RNA Sequence-Structure Co-design

Abrar Rahman Abir, Liqing Zhang

1 Hyperparameter Details

The hyperparameter details are presented in Table S3.

2 Performance Comparison on Independent PRBAB1.0 dataset

To further demonstrate the robustness and generalizability of RNA-EFM, we evaluated our method on the PRBAB1.0 dataset as shown in Table S1, which comprises 73 protein–RNA complexes entirely independent from both the training set and the RF2NA split. Across all core metrics including RMSD, lDDT, native sequence recovery, and IMP, RNA-EFM consistently outperformed competing approaches, achieving substantial improvements over both RNAFlow and MMDiff. These results highlight the superior ability of RNA-EFM to generate accurate and biophysically realistic RNA designs even on unseen complexes, confirming its effectiveness for practical RNA–protein engineering scenarios.

Table S1: RNA structure and sequence generation results on the PRBAB1.0 dataset. We report Mean ± Standard Error of the Mean (SEM) for RMSD, lDDT, native sequence recovery, and IMP (%).

**Method**	**RMSD**	**lDDT**	**Sequence Recovery**	**IMP (%)**
Conditional MMDiff	15.87 ± 1.22	0.39 ± 0.03	27.16 ± 1.39	41.74 ± 2.15
RNAFlow-Base	15.02 ± 0.77	0.48 ± 0.02	31.04 ± 1.23	47.88 ± 1.66
RNAFlow-Traj	15.39 ± 1.12	0.50 ± 0.01	32.29 ± 1.07	49.61 ± 1.93
RNAFlow-Base + Rescore	14.64 ± 0.95	0.51 ± 0.03	33.15 ± 0.98	50.73 ± 1.58
RNAFlow-Traj + Rescore	15.61 ± 1.34	0.47 ± 0.02	30.84 ± 1.42	46.37 ± 1.91
**RNA-EFM**	**14.10 ± 0.69**	**0.56 ± 0.02**	**38.89 ± 1.11**	**57.91 ± 1.05**

3 Sequence Generation Performance Comparison Based on Levenshtein (edit) distance

Table S2: Mean ± Standard Error of the Mean (SEM) Levenshtein (edit) distance between predicted and ground-truth RNA sequences. Lower is better.

**Method**	**RF2NA Split**	**Sequence Similarity Split**
LSTM	25.7 ± 0.5	29.1 ± 0.8
Conditional MMDiff	27.4 ± 0.8	28.8 ± 0.9
RNAFlow-Base	19.2 ± 0.5	21.8 ± 0.7
RNAFlow-Traj	18.6 ± 0.6	22.4 ± 0.6
RNAFlow-Base + Rescore	17.5 ± 0.7	21.1 ± 0.8
RNAFlow-Traj + Rescore	16.9 ± 0.9	20.8 ± 0.9
**RNA-EFM**	**12.8 ± 0.4**	**15.9 ± 0.5**

To further benchmark sequence generation accuracy, we report the Levenshtein (edit) distance between predicted and ground-truth RNA sequences for all methods. As shown in Table S2, RNA-EFM achieves the lowest edit distance in both the RF2NA and sequence similarity splits, demonstrating improved sequence fidelity over all baselines. This metric complements the sequence recovery rate and provides an additional, widely used measure of nucleotide-level similarity.

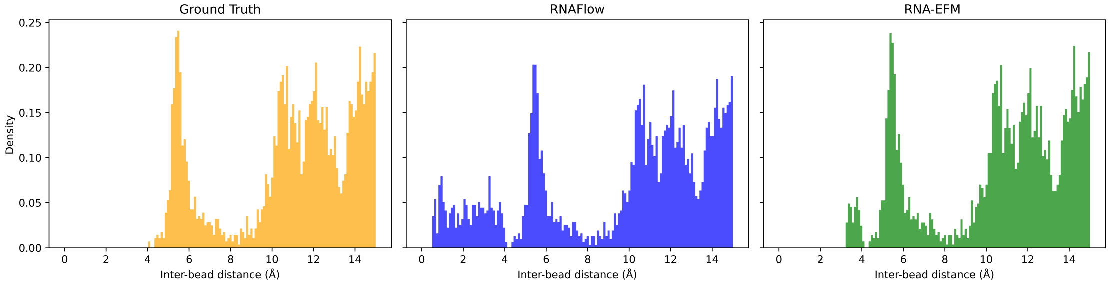

Figure S1: Inter-bead distance distributions for ground truth RNA structures, RNAFlow, and RNA-EFM.

4 Role of Lennard-Jones Potential in Clash Prevention

To quantitatively validate the role of the Lennard-Jones potential in our energy minimization procedure, we analyzed the inter-bead distance distributions of generated RNA structures. As shown in Figure S1, RNAFlow predictions exhibit a substantial population of steric clashes at short distances (< 2.0 Å), indicating physically unrealistic atomic overlaps. In contrast, RNA-EFM predictions, enhanced with LJ potential-based refinement, effectively eliminate these short-distance clashes, producing a distance distribution that closely approximates the native RNA profile. These results demonstrate that the primary function of the LJ

Table S3: RNA-EFM hyperparameters and configuration settings

**Parameter**	**Value**	**Description**
seed	42	Random seed for reproducibility
save	True	Whether to save model checkpoints
data_path	./data/	Data directory (preprocessed and raw)
process_raw	True	Process datasets from raw .pdb files
save_processed	True	Save processed datasets
top_k	10	Number of k-nearest neighbors
num_rbf	16	Number of radial basis functions
num_posenc	16	Number of positional encodings
num_conformers	3	Number of conformers per sequence
node_in_dim	[64, 4]	Node feature input dimensions (scalar, vector)
node_h_dim	[128, 16]	Node feature hidden dimensions
edge_in_dim	[32, 1]	Edge feature input dimensions (scalar, vector)
edge_h_dim	[32, 1]	Edge feature hidden dimensions
num_layers	4	Number of encoder/decoder layers
drop_rate	0.1	Dropout rate
out_dim	4	Output dimension (4 RNA bases)
max epochs	100	Number of max training epochs
lr	0.001	Learning rate
batch_size	8	Batch size
max_nodes	5000	Maximum nodes per batch
num_workers	8	DataLoader workers
val_every	5	Validate every N epochs
ϵ	0.2	Depth of LJ potential well
σ	3.5 Å	Distance where LJ potential is zero
min_distance	0.5 Å	Minimum distance for stability
Structure loss	MSE	Mean squared error on backbone
Sequence loss	Cross Entropy	Cross-entropy for nucleotide recovery
Train ratio	50% FM / 50% refinement	Ratio of alternating training phases

potential is to prevent steric clashes that would otherwise occur during inference. RNA-EFM generates structurally plausible and thermodynamically stable RNA conformations.
